# Effect of Two Immediate Dentin Sealing Approaches on Bond Strength of Lava™ CAD/CAM Indirect Restoration

**DOI:** 10.3390/ma14071629

**Published:** 2021-03-26

**Authors:** Hassan Faez Abdullah Gailani, Cristina Benavides-Reyes, María Victoria Bolaños-Carmona, Eva Rosel-Gallardo, Purificación González-Villafranca, Santiago González-López

**Affiliations:** 1 Department of Operative Dentistry, School of Dentistry, University of Granada, Campus de Cartuja, Colegio Maximo s/n, E-18071 Granada, Spain; hassanfaez@correo.ugr.es (H.F.A.G.); sglopez@ugr.es (S.G.-L.); 2 Department of Pediatric Dentistry, School of Dentistry, University of Granada, Campus de Cartuja, Colegio Maximo s/n, E-18071 Granada, Spain; mbolanos@ugr.es; 3 Master Odontología Multidisciplinar y Estética, University of Granada, Avenida de Fuentenueva s/n, E-18071 Granada, Spain; erosel@ugr.es (E.R.-G.); purichi85@hotmail.com (P.G.-V.)

**Keywords:** IDS, DDS, universal adhesives, self-adhesive cements, CAD/CAM restoration

## Abstract

The objective of this work was to compare the micro-tensile bond strength (µTBS) of CAD/CAM (Computer-Aided Design/ Computer-Aided Manufacturing) specimens cemented with different pairing of adhesives and resin-cements using two Immediate Dentin Dealing (IDS) approaches in comparison with Delay Dentin Sealing (DDS). Coronal dentin from 108 molars were divided into nine groups (*n* = 12) depending on the adhesive/resin-cement (A-C) assigned. Lava™ Ultimate (4 × 10 × 10 mm) was cemented according to different strategies: IDS1(cementation after dentin sealing), DDS (dentin sealing and cementation at 2-weeks), IDS2 (immediate dentin sealing and cementation at 2-weeks). Samples were sectioned and tested until failure to determine the µTBS. Failure mode was categorized as dentin/cement (DC), at Lava™ Ultimate/cement (LC) and hybrid (H). Kruskal–Wallis and Mann–Whitney U tests and influence of the type of failure on the µTBS by survival analysis with competing risk was explored. Mostly, µTBS values were equal or higher in IDS2 than DDS. In general, A-Cs that showed higher µTBS, have high percentages of LC failure. Survival analysis with competing risk between DC + H and LC values showed that some A-Cs would significantly increase the µTBS values for IDS2. A-Cs with the highest adhesion values showed a high percentage of fractures at the LC interface, suggesting that the adhesion at the adhesive/dentin interface would be higher.

## 1. Introduction

In restorative dentistry, usually compromised larger posterior cavities either biomechanically or aesthetically have to be restored using partial indirect restorations and adhesive technology. Its clinical success depends on factors such as the composition of the indirect restoration material and the adhesive cementation procedure [1,2,3].

The Immediate Dentin Sealing (IDS) technique consists of the application of dental bonding agent immediately after tooth preparation and before impression taking, instead of Delay Dentin Sealing (DDS) that represents a common clinical practice where the dentin adhesive is applied just before cementing the restoration in a second visit [4]. It has been reported that IDS protect freshly cut dentin against contamination. In addition, collagen of the hybrid layer is guarded against collapsing, and subsequently, the bonding procedures of indirect restorations result in higher bond strength values [5], improving marginal sealing, reducing post-cementation sensitivity, and improving cavity adaptation of indirect restoration. It also increases patient comfort during the provisional restoration stage with limited need for anesthesia in the cementing appointment of definitive restoration [6,7,8].

The chairside CAD/CAM (Computer-Aided Design/Computer-Aided Manufacturing) technique is increasingly used in dentistry. There is a variety of available CAD/CAM restorative material, as well as different types of ceramic, composite resin, and hybrid materials, with which we are able to produce high-quality indirect restorations. An additional advantage of the CAD/CAM technique is the possibility of cementing the indirect restoration in the same visit [9]. One of the most used materials of this type is Lava™ Ultimate CAD/CAM block, a nano-ceramic resin composite containing approximately 80% by weight of nanoceramic particles bound in a resin matrix, with a dentin-like modulus with an elasticity of 12.8 GPa [10]. When this technology is not available in the dental clinic, indirect restoration requires the use of provisional restoration. The influence of provisional restoration residue on bond strength to dentin in adhesive cementation is unclear. Some studies suggest that the provisional restoration may affect the bond strength, while the other studies suggest that only provisional cements with eugenol can affect bond strength [11]. On the other hand, Hayashi et al. [12] reported that the IDS restoration without temporary restoration produces maximum bond reliability and ensuring durability against debonding.

An adhesive procedure is an important step for the longevity of indirect restorations [13]. Etch-and-rinse adhesive agents have been proposed for the IDS technique, nonetheless, it also has been used the latest self-etch and universal adhesives to enhance bond strengths [5]. In the IDS technique, the dentine bonding agent reacts with oxygen and forms a thin, soft, and sticky superficial un-polymerized layer called the Oxygen Inhibition Layer (OIL) [14,15], and it is created by an increasingly low conversion rate of the resin. It can react with the impression material avoiding its polymerization and also most provisional restorative materials can bond to the IDS surface preventing its easy debonding [14]. In order to avoid that, it has been recommended to use glycerin gel to prevent the formation of OIL [14].

It is yet unknown which method is the most suitable for conditioning dentin treated with IDS prior to adhesive cementation [1]. It has been reported that soft air abrasion, airborne particle abrasion with Al_2_O_3_, or fluoride-free pumice paste systems resulted in the highest bond strength. The abrasive process eliminates contaminant layers, and the roughness increases the surface area for bonding surface, supplying some degree of mechanical interlocking with the adhesive [13].

In this work, different clinical situations have been simulated, intended to assist the clinician in making decisions when an indirect restoration with Lava™ is performing: (1) cavity preparation, dentin sealing, and cementation of the indirect CAD/CAM restoration is performed in the same step; (2) cavity preparation and a provisional restoration in the first step followed by a second step for dentin sealing and cementation of CAD/CAM restoration; and (3) cavity preparation, immediately dentin sealing, and provisional restoration in the first step followed by a second step for cementation of CAD/CAM restoration.

The purpose of this work was to compared the adhesion to dentine (micro-tensile bond strength (µTBS)) of Lava™ Ultimate CAD/CAM restorations cemented with eight universal adhesives and a three-step etch-and-rinse adhesive and their corresponding resin cements using two IDS approaches in comparison with DDS. The null hypothesis of this study was that there is no significant difference in the µTBS of different universal adhesives with their corresponding resin cement nor between the different clinical strategies (IDS or DDS) when Lava™ Ultimate CAD/CAM restorations were adhesively cemented to the dentin.

## 2. Materials and Methods

The current research was approved by the Ethics Committee of the University of Granada (Spain) (#1005/CEIH/2019).

### 2.1. Sample Preparation

One hundred and eight sound human molars extracted for periodontal reasons were cleaned and stored in a solution of 0.1% thymol until sample preparation. The roots of all teeth were removed by a slow-speed cutting machine (Struers, Copenhagen, Denmark) under water irrigation 2 mm below the cement enamel junction. Then, pulp chambers were cleaned using 2.5% sodium hypochlorite. Samples were connected to a simulated pulp pressure (SPP) system by a previously described method [16] maintaining the tooth under pressure and humidity conditions throughout the whole experiment. The occlusal coronal third of the crown was removed by a slow speed cutting machine (Struers, Copenhagen, Denmark) at 300 rpm to exposed the dentine surface and finished with 600 grit SiC paper under water [17,18].

Samples of 4-mm thickness ×10-mm width ×10-mm length were cut from CAD/CAM Lava™ Ultimate blocks (3M ESPE, St. Paul, MN, USA) by a slow-speed cutting machine under water cooling in order to obtain a uniform surface for adhesion.

**Figure 1:**
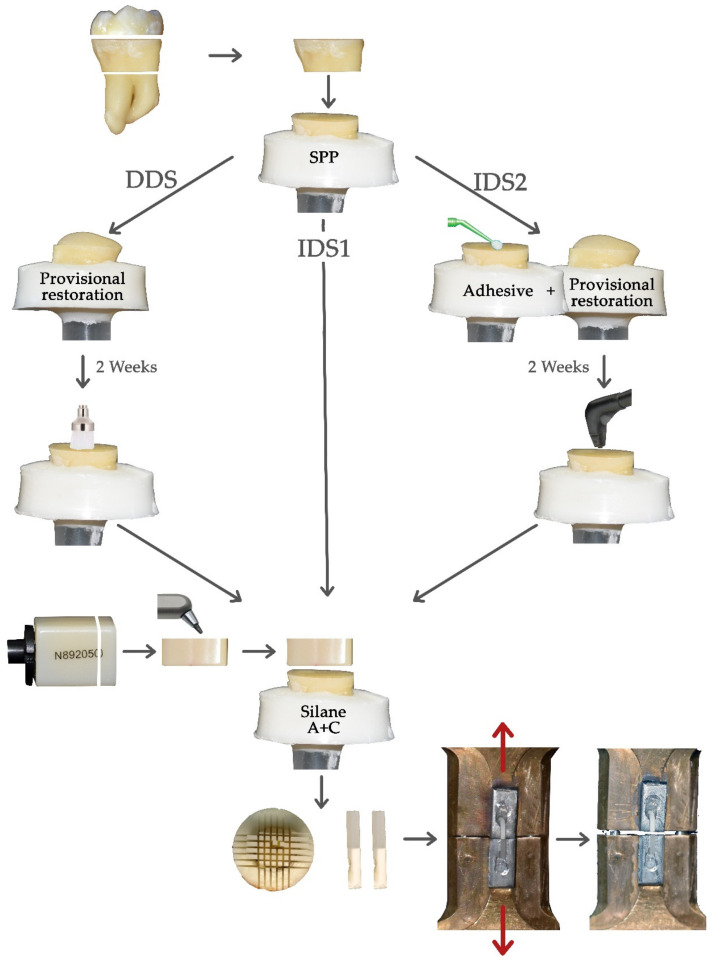
Sample preparation and analysis flow in the different groups. SPP: Simulated Pulp Pressure; A + C: Adhesive + Cement; DDS: Delay Dentin Sealing; and IDS: Immediate Dental Sealing.

Specimens were randomly divided into nine groups (*n* = 12) depending on the pairing of dentin adhesive and resin cement (A-C) assigned. Then each group was divided into 3 subgroups (*n* = 4), for each dentin sealing strategy (Figure 1):

1. Protocol 1 (IDS1): Adhesive bonding agent was first applied on each specimen and then a specimen of Lava™ Ultimate was cemented to flat dentin surfaces with the corresponding resin cement.
2. Protocol 2 (DDS): Occlusal dentin surface of each specimen was covered with a layer of 3-mm provisional restoration material (Telio CS onlay, Ivoclar Vivadent, Schaan, Liechtenstein) and polymerized for 15 s and, subsequently, the specimens were left under SPP at room temperature. After two weeks, the temporary filling was removed and the dentin surface was cleaned with a polishing brush under water irrigation and finally, dentin adhesive was applied and a sample of Lava™ Ultimate was cemented with the corresponding resin cement.
3. Protocol 3 (IDS2): Adhesive bonding agent was applied immediately after preparation and photopolymerized. Then the adhesive layer was covered with glycerin gel and cured yet another 10 s in order to prevent the OIL and, finally, the occlusal dentin was covered by 3 mm of provisional restoration material like the protocol 2. After two weeks, the temporary filling was removed and the occlusal dentin was sandblasted with PROPHY MATE cleaning powder “Calcium Carbonate” with Perio-Mate (NSK, Kanuma, Tochigi, Japan) for 1 min and a new adhesive layer was applied and polymerized [4]. Finally, a sample of Lava™ Ultimate was cemented with the corresponding resin cement.

All universal adhesives were applied brushing the dentine surface and following the manufacturer’s recommendations. Adhesives were polymerized using the lamp Bluephase (Ivoclar Vivadent, Schaan, Liechtenstein) output 1200 mW/cm^2^. The brand, composition, and manufacturer details of each adhesive and its corresponding cement (A-C) are shown in Table 1.

Before luting, the surface of each Lava™ Ultimate specimen was sandblasted (Bio-Art Microblaster lab) with 50 µm particles Al_2_O_3_ for 2 min, ultrasonically cleaned for 5 min in distilled water, and air-dried. Silane RelyX™ Ceramic Primer (3M ESPE) was applied for 1 min according to the silane manufacturer’s instructions, afterwords, a new layer of adhesive was applied and the solvent evaporated and it was left uncured. Each Lava™ specimen was cemented on the dentin substrate with the corresponding resin cement. The luting procedure was performed under a constant pressure of 1 kg (0.098 MPa) by means of a metal tool until the seating of the material was complete. The seating force was applied for the first 5 min leaving the material to set in the self-curing mode [19], and being photopolymerized from the top for 40s with an LED curing unit Bluephase (Ivoclar Vivadent, Schaan, Liechtenstein). Finally, specimens were left under SPP at room temperature for 24 h. 

### 2.2. Micro-Tensile Bond Strength (µTBS)

Subsequently, specimens were vertically sectioned into bars of 1 × 1 mm with a hard tissue cutting machine Accutom-50 (Struers, Copenhagen, Denmark). The thickness of each stick was assessed by means of a digital caliper. Specimens were glued with cyanoacrylate gel (Superglue 3 Gel, Loctite, Henkel, Düsseldorf, Germany) to a unitary gripping device. Samples were assayed in a universal tester Instron 3345 (Instron, Norwood, MA, USA) at a crosshead speed of 0.5 mm/min until failure (Figure 1).

### 2.3. Failure Analysis

Fractured surfaces were inspected under magnification (40×) with a stereomicroscope (Olympus SZ60, Tokyo, Japan) to determine the mode of failure. These failures were classified as cohesive dentin failure (D), at interface dentin/resin cement (DC), at interface Lava™ Ultimate/resin cement (LC), and hybrid (H) mixed between both surfaces following the Academy of Dental Materials guidance [17]. For statistical analysis D failures were discarded and just DC and H failures were selected, since they represent the real adhesion of the dentin adhesive interface. The µTBS values in the LC failures would considerably higher than those reported since the adhesive/dentin interface is more resistant in these cases. Also, pretesting failure (PT) specimens were included in the statistical analysis with the lower adhesive value of their experimental groups, as recommended by the Academy of Dental Materials [17].

Additionally, representative specimens from each group were mounted in aluminum holders, gold sputter-coated (Polaron E-5000, Polaron Equipment, Watford, UK), and studied in a Zeiss DMS 950 scanner electron microscope (Zeiss DSM 950, Carl Zeiss, Germany) at 150 and 500× magnification.

### 2.4. Statistical Analysis

Means and standard deviations were calculated for each of the parameters analyzed. Normality of data distribution was tested using the Kolmogorov–Smirnov and Shapiro–Wilk tests. Because a normal data distribution was not found, non-parametrical tests were used. Comparisons between the different adhesive/resin cements at the same adhesive protocol were performed using Kruskal–Wallis and Mann–Whitney U tests. Likewise, the comparisons between the different adhesive protocols were made with Mann–Whitney U and Kruskal–Wallis tests. Relative frequencies of failure types were provided and the χ2 test was used to detect group differences. Finally, the influence of the type of failure on the µTBS by survival analysis with competing risk and Mann–Whitney U test were explored. All statistical analyses were performed using the software SPSS 24.0 (SPSS Inc., Chicago, IL, USA) and OriginPro 2019b (OriginLab Corporation, MA, USA). A level of significance of *p* < 0.05 was established.

## 3. Results

A Kruskal–Wallis multiple comparisons test showed significant differences between A-C (χ2 = 113.310; *p* < 0.001) and dentin sealing protocols (χ2 = 12.835; *p* < 0.005). 

Means, standard deviations, and pairwise comparisons performance using Mann–Whitney U, number of beams, and percentage of each failure type are shown in Table 2. Kerr, Dentsply, and Ivoclar performed well reaching the highest µTBS values in the three protocols studied. Bisco D, Kerr U, and Voco obtained, in general, the worse results. The highest value was obtained by 3M in IDS2 and the lowest by Bisco D in IDS1. 

Comparing the adhesive protocols within each A-C pairing, Kerr, Kerr U, and Dentsply did not show statistically significant differences. IDS1 protocol showed intermediate values in 3M, Voco, Bisco D, and Bisco U without showing statistically determined differences with the other protocols, but differences between DDS, with the lowest values reached, and IDS2 were found. Ivoclar and Coltene follow the same trend obtaining IDS1 the highest values of µTBS and the lowest with DDS, this difference being statistically significant in the case of Coltene.

In addition, it was observed that values of the µTBS in IDS2 were higher than in DDS for most of them, although these differences only showed statistical significance for 3M, Voco, Bisco D, and Coltene. Results also showed that 3M, Voco, Bisco D, Bisco U, and Coltene have a similar behavior, with higher bond values in IDS1 and IDS2 and lower values in the DDS protocol. A-Cs without significant differences between IDS2 and DDS strategies reached, in general, the highest µTBS values. Bisco U was the only one where DDS values were statistically significantly higher than IDS2.

Comparing Immediate Sealing Protocols, it was observed that no pairs of A-Cs showed statistically significant differences between IDS1 and IDS2 except Ivoclar where IDS1 obtained significantly higher µTBS values.

**Figure 2:**
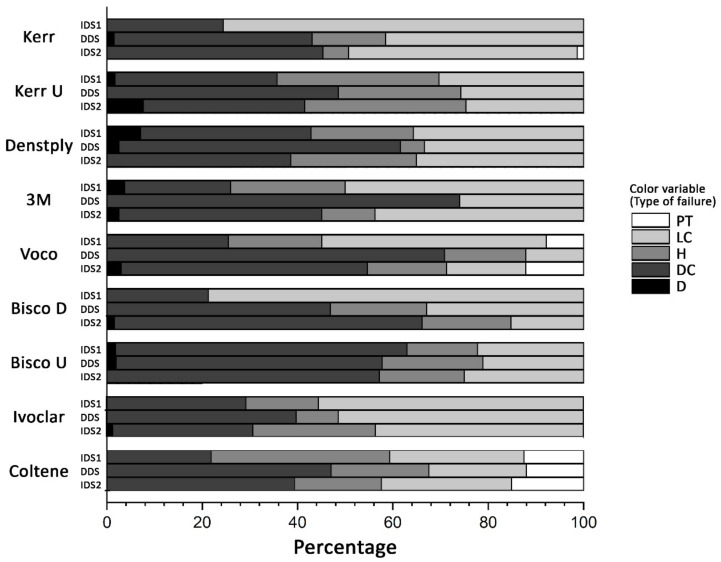
Failure mode analysis (percentage). type of failure: pretest (PT), at Lava™ Ultimate/resin cement interface (LC), at interface dentin/resin cement (DC) and hybrid (H) and dentin failure (D).

Figure 2 shows the failures type for each A-C, and Table 2 specifies the number of specimens and the percentage. Analyzing these values, it is observed that there are a high percentage of failures in the LC interface, which would be 46.1% for IDS1, 30.1% for DDS, and 32% for IDS2. These high percentages of failures in the LC interface usually occur in A-Cs with higher µTBS values, such as Kerr, 3M, and Ivoclar, except for Bisco D which was 78.7% in the IDS1 protocol.

**Figure 3:**
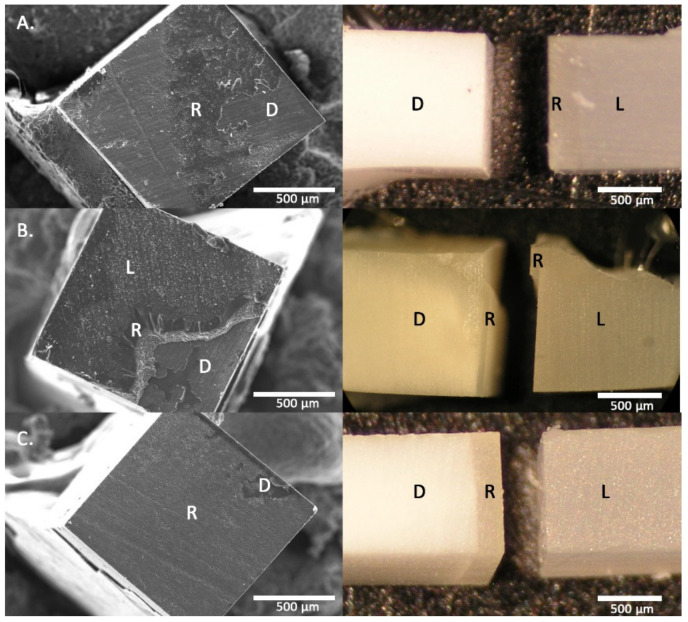
Representative images of the different types of failures as seen by scanning electron microscopy (SEM) (**left**) and stereomicroscope (**right**). (**A**) dentin/resin cement (DC); (**B**) Hybrid failure; (**C**) Lava™ Ultimate/resin cement (LC). R = resin cement; D = dentin; L = Lava™ Ultimate. Scale bar: 500 μm.

Statistical significance between the µTBS average of the DC + H failures and the LC values had been explored. Survival analysis with competing risk demonstrated that the LC failures could compete with the adhesive ones (DC + H). Table 2 shows the comparison between µTBS values of DC + H and LC failures and Figure 3 shows representative images of the different types of failures. Kerr significantly would increase the µTBS values for the three protocols in the DC + H interface because 40% of failures occur in the LC interface. 3M and Ivoclar would increase the µTBS values for DDS and IDS2. Dentsply for the IDS1 and Coltene for IDS2, would agree in all these groups with high values of µTBS and high percentage of fractures in the LC interface. A-C pairings with low µTBS did not expect higher adhesion values except for Bisco U in IDS1 and IDS2.

## 4. Discussion

The main hypothesis of this study was partially rejected because there were significant differences among µTBS between A-C materials and between IDS1, IDS2, and DDS strategies when Lava™ Ultimate CAD/CAM restorations were adhesively cemented with some universal adhesives and its corresponding resin luting agent.

Recently, machinable ceramic, composite, and hybrid restoratives systems yield a satisfactory restoration with an acceptable marginal adaptation and clinical longevity. The IDS technique has been suggested as an alternative to improve the quality of adhesion for indirect restorative procedures since it provides numerous advantages for the patient and longevity of the restoration with respect to the DDS [1,4,6,7,8,20,21,22,23]. Clinicians have two different clinical strategies for IDS depending on the availability or not in the CAD/CAM technology in the clinic. If CAD/CAM restorations can be performed in the clinic, the delay time between the IDS and the cementation of the restoration is the time it takes to process and cement the CAD/CAM restoration and it is the strategy used in IDS1. Otherwise, clinicians must send the restoration to the laboratory, leaving a waiting time between the IDS and the cementation of the restoration, we have explored this possibility in IDS2. DDS groups represent a common clinical practice where the adhesive resin layer is applied just before luting adhesively the restoration in a second visit.

The results of this research are highly dependent on A-C showing great variability in µTBS values and between IDS1, IDS2, and DDS. In the present study, the three-step etch-and-rinse adhesive Optibond FL was chosen as the gold standard because this adhesive is known for its high filler load and high mechanical strength resulting in higher µTBS [1,24]. Cementing of Lava™ Ultimate specimen with Kerr has obtained high values of µTBS in the three protocols studied, without any differences between them. Adhesion values have been shown to be primarily material dependent. Dentsply and Bisco U have obtained high µTBS values in the three protocols studied statistically similar to Kerr, although from a clinical point of view it would be recommended to use IDS techniques due to the additional advantages that this clinical approach brings over DDS. 3M and Coltene obtained adhesive values similar to previous A-Cs, except for DDS protocol where µTBS values were lower, these results are in accordance with those reported by most authors who report better results when IDS2 is performed [4,14,20,21,22,25,26,27]. µTBS values obtained by 3M in the IDS2 protocol were the highest, statistically significant, compared to the other A-C. This behavior may be due to the optimization of this universal adhesive, since Relyx Ultimate cement and Lava™ Ultimate are 3M products, being in agreement with this point [28]. Most of the reviewed publications agree that µTBS values with IDS2 were better than those with DDS [4,7,12,29]. Our results have shown that most of the adhesives/resin cements obtained higher µTBS values with IDS2 than DDS, although they were only significant for 3M, Voco, Bisco D, and Coltene although with great differences between the adhesion values obtained. Generally, A-Cs that have obtained the highest adhesion values do not show significant differences between the three protocols or behave better with the IDS protocols. This variability may be due to the fact that in our methodology we have used universal adhesives with their corresponding resin cements, which is what is usually done in the clinical routine.

Self-adhesive resin cements simplify clinical procedures and overcome the technique sensitivity of multistep systems. These resin cements do not require any pretreatment of the tooth surface, and their application is accomplished through a single clinical step [19,30]. In the three protocols studied, the cements were directly applied on the previously light-cured universal adhesive, although adhesion between the dentin adhesive layer and resin cement will be effective due to the presence of unreacted methacrylate groups present in the adhesive layer and the resin cement [26], there are additional characteristics that can influence the adhesion values, such as the pH of the used universal adhesives, which mostly have ultra-mild pH (between 2.5 and 3.2) except Future bond Universal single bond, which has mild pH (2.3) [31]. The main constituents of self-adhesive resin cement are functional acidic monomers (e.g., MDP, BMP, Penta-P, 4-META, etc.), conventional di-methacrylate monomers (e.g., bis-GMA, UDMA, and TEGDMA), filler particles, and activator–initiator systems [32], residual acidic monomers can have an impact on the polymerization reaction of the cement, especially by inhibiting the action of the amine accelerator required for the camphorquinone–amine photo initiator system present in essentially all current cement systems [33]. All of these factors and the disparity in monomer amount in each resin cement formulation can influence the differences found between the different groups of adhesive/resin cements. It should be noted that in the comparison of IDS1 with IDS2 within each pair of A-Cs, both have the same performance from the statistical point of view, except for Ivoclar where IDS1 was higher than IDS2. This reinforces the recommendation for the use of IDS in the clinic.

We needed to consider two different interfaces in the bond between indirect CAD/CAM restorations and the tooth structure: the one established between dentin/enamel and the resin cement, and the one between resin cement and CAD/CAM restorations. Most of the articles use all types of failure evaluated to calculate the µTBS means [4,9,26,29], however, in this work a high percentage of fractures in the LC interface has been found, ranging from 22.2–75.5%. The high number of specimens (*n* = 1595) has allowed discarding cohesive failures in D and LC interface, and only used adhesive (DC) and mixed (H) failures for the calculation of the averages, since they are the ones that truly reflect the resistance of the adhesive interface to dentin. Later, we have compared this mean with that obtained with the values at the LC interface, assuming that when this type of failure occurs it is because the resistance of the adhesive interface is more resistant and it could be expected that the real values of the adhesion were higher than the values of adhesive failure between A-C and Lava™ Ultimate. Most of the adhesive/resin cements pairing that have statistically high adhesive bond values, have high percentages of failures in the LC interface as Kerr in the three protocols or in some of the protocols such as 3M and Ivoclar in DDS and IDS2 protocols, Dentsply in IDS1 protocol, Coltene in IDS2, and Bisco D in IDS1and IDS2. It is important to know which of these interfaces should be optimized because the weakest one will determine the final bond strength of the cemented restoration [34]. Lava™ Ultimate is a composite formed of a resinous matrix highly filled with silica and zirconia particles, and presents a higher degree of conversion [35]. It has been reported that the conditioning of the surface in the adhesive cementation of Lava™ Ultimate with airborne-particle abrasion leads to the higher adhesive strength and micromechanical retention [36], as also recommended in the manufacturer’s instructions, as the procedure that was carried out in this work. In addition, universal adhesive has been applied, most of which includes in their chemical composition methacrylic monomers—silane or phosphate monomers, that allow them to prime metal, silica-based ceramic, and zirconia restorations for improved adhesion [36]. On the other hand, Peumans et al. [37] has reported that when Lava™ Ultimate was cemented with self-adhesive cement, it obtained significantly greater bond strength than conventional resin cement. The results of our study highlighting the need to improve the interface with Lava™ Ultimate and, thus, be able to assess the true strength of dentin adhesion of some of the Adhesives/resin cement studied. Also, it suggests further research to improve the adhesion milling Lava™ Ultimate by the action of the diamond burs because this process increased the roughness of all CAD-CAM ceramic and composite resin blocks [38], therefore, better performance of this interface could be expected in a real clinical situation.

Kerr U obtained statistically lower µTBS values than the control group in the three protocols studied, although there were no significant differences between the three protocols. It should be noted that the percentage of failures in the LC interface was lower, which would explain the absence of statistically differences between the average of the adhesive interface (DC + H) and LC.

In Voco and Bisco D, DDS was the protocol that obtained the lowest values, where Bisco D pairing obtained the worst values in the three protocols. This may be due to residual acid monomers from these adhesives required for dual polymerization have shown to negatively affect the degree of cure of dual-cured materials, since they seem to interact chemically with the amine initiator that dual-cured resins contain [33]. It may also have been influenced by Duo-Link cement not being a self-adhesive cement and, therefore, has no active monomers in its composition.

Remnants of the provisional cements used to lute provisional restorations have been demonstrated to influence the bond strength of the final restoration. Various authors have evaluated methods for the removal of provisional cement in vitro. Telio resin temporary filling in DDS and IDS2 for two weeks has been used in this work and that was placed without any intermediate cement, which facilitated the cleaning of the dentin before bonding. Although some authors recommend the use of prophylactic, fluoride or pumice paste with a rotary low-speed brush, most of them recommend sandblasting the adhesive layer in Immediate Dental Sealing. This sandblasting results of large area of dentin are exposed, for this reason, Prophy Mate (soft air erosion) has been used. When calcium carbonate was used for airborne-particle abrasion, the surface roughness increased substantially with minimal abrasive effect on the adhesive layer [39]. In Bisco U, Kerr U, 3M, Coltene, Voco, and Bisco D pairing, the dentin bond values were higher in the IDS2 protocol than in the DDS, although only in the last four pairs was it significant, this may be because remnant debris could have remained on the dentin surface. The application of temporary restorations did not affect the bond strength when IDS2 protocol was applied.

Positive intra-pulpal pressure (SPP) that is one of the factors able to negatively interfere with dentin adhesion, reducing bond strengths values [19,26,40,41]. SPP has been used in all protocols and the percentage of pretest failures has been relatively low (<3%) [26] suggesting that the use of the IDS technique is effective in promoting greater bond strength values and reduced nanoleakage patterns in indirect restorative procedures, especially in the presence of SPP. It has also been reported that the adhesive interface obtained by using IDS was stable over time, they did not find significantly differences between the one-week and six-month water storage data [1].

This study had some limitations. The influence of different factors on the success and survival of restorations has been studied. This success is affected not only by the materials, but also by the clinical conditions (skill of the clinician, infection, and general health of the patient) [42]. Thus, results should be interpreted with caution when extrapolating them to clinical conditions for different reasons. First, the internal surface is achieved by cutting at a low speed, and not by milling at the CAM procedure. Also, this study is a statistical study, being desirable to study the dynamic behavior of hybrid materials such as Lava™ as Khosravani (2019) [43] has shown that progressive dynamic loading leads to a smoother surface and the surface roughness affected the hardness of the specimen. Therefore, more clinical studies are necessary.

## 5. Conclusions

Within the limitations of this in vitro study, it can be concluded: Universal adhesive/resin cements values of µTBS are mainly material dependent. Pairing of three-step etch-and-rinse Kerr and Universal Adhesives Dentsply, Ivoclar, 3M, and Coltene obtained the highest values of adhesion. Mostly µTBS values were equal or higher in IDS2 than DDS. Adhesives/resin cements with the highest adhesion values showed a high percentage of fractures at the Lava™ Ultimate/cement interface, suggesting that the adhesion values at the adhesive/dentin interface would be higher. 

## Figures and Tables

**Table 1:** Manufacturer and main chemical composition of the different products used in this study.

Study Group Manufacturer	Adhesives (Batch Number)	Chemical Composition	Cements (Batch Number)	Chemical Composition
Kerr (Kerr, Orange, CA, USA)	OptiBond FL pH = 2.0 (84018)	HEMA, GPDM, PAMM, ethanol, water, photoinitiator Adhesive: TEGDMA, UDMA, GPDM, HEMA, bisGMA, filler, photoinitiator.	Maxcem Elite cement (6504758)	19–40% Methacrylate ester monomers, other-inert mineral fillers activators stabilizers, colorants, YF.
Kerr U (Kerr, Orange, CA, USA)	OptiBond Universal pH = 2.5–3 Ultra-Mild (6769529)	37.5% H_3_PO_4_, BisGMA, GPDM, HEMA, PAMM, barium glass, silica, sodium, hexafluorodilicate, ethanol.	Maxcem Elite cement (6504758)	19–40% Methacrylate ester monomers, other-inert mineral fillers activators stabilizers, colorants, YF
Dentsply (Dentsply, Konstanz, Germany)	Prime and Bond active universal pH = 2.5 Ultra-Mild (181000042)	Phosphoric acid modified acrylate resin, multifunctional acrylate, bifunctional acrylate, isopropanol, initiator, stabilizer.	Calibra Ceram Adhesive Resin Cement (1710171)	Urethane di-methacrylate, Di-and tri-methacrylate resin, phosphoric acid modified acrylate resin, Barium Born Fluro-Alumino Silicate Glass, Organic Peroxide initiator, CQ, Phosphone Oxide Photoinitiator, Accelerators, butylated Hydroxy Toluene, UV Stabilizer, Titanium Dioxide, Iron Oxide, Hydrophobic Silicon Dioxide, Particles of inorganic filler range from 16 nm to 7 µm, average particle size 3.8% µm, total filler 46.3% vol.
3M (3M St Paul, MN, USA)	Scotchbond Universal Adhesive pH = 2.7 Ultra-Mild (70918A)	MDP, dimethacrylate resins, HEMA, methacrylate-modified Polyalkenoic acid copolymer, Filler, Ethanol, Water, Photo initiators, Silane	Relyx Ultimate Adhesive Resin Cement (3472645)	Base paste: methacrylate monomers, radiopaque alkaline (basic) fillers, initiator, stabilizer, rheological additives Catalyst paste: methacrylate monomers, radiopaque alkaline (basic) fillers, initiator, stabilizer, pigments, rheological additives, fluorescence dye, dark cure activator.
Voco (Voco, Cuxhaven, Germany)	Future bond Universal single bond pH = 2.3 Mild (1807614)	Organic acid, UDMA, HEMA, CQ, BHT	Rebilda DC Cement (1704534)	BisGMA, UDMA, DDMA, BHT, Dibenzoyl peroxide, CQ, Silica, barium borosilicate glass ceramic., accelerators.
Bisco D (Bisco Universal, Schaumburg, IL, USA)	Universal Primer Dual Cured Adhesive pH = 3.1 Ultra Mild (1600358474)	Primer A: Acetone, Ethanol, Na-N-totygycine glycidylmethacrylate. Primer B: Acetone, Ethanol, Biphenyl dimethacrylat.	Duo-Link Universal (1600358244)	Base: BisGMA, TEGDMA, UDMA, Glass filler Catalyst: BisGMA, TEGDMA, Glass filler
Bisco U (Bisco Universal, Schaumburg, IL, USA)	All Bond Universal pH = 3.1–3.2 Ultra Mild (1800002797)	BisGMA (5–30%) TEGDMA (5–20%) Glass filler (5–80%)	Duo-Link Universal (1600358244)	Base: BisGMA, TEGDMA, UDMA, Glass filler Catalyst: BisGMA, TEGDMA, Glass filler
Ivoclar (Ivoclar vivadent, Schaan, Liechtenstein)	Adhese Universal pH = 2.5–3 Ultra Mild (W41872)	Methacrylates, ethanol, water, highly dispersed silicon dioxide, initiators and stabilizers.	Variolink (W95570)	Dimethacrylates, Adhesive, monomer, Filers, Initators, Stabilizers.
Coltene (Coltene, Cuyahoga Falls, OH, USA)	One coat7 Universal pH = 2.8 Ultra Mild (H39695)	Methacrylates including 10-MDP, photoinitiators, ethanol, water	Solocem cement (SC)	UDMA, TEGDMA, 4-META, 2-HEMA, DBP; BP
Purified water, glycerin, methylparaben, propylparaben, propylene glycol, hydroxyethylcellulose, disodium phosphate, sodium phosphate, tetrasodium EDTA.				
3M St Paul, MN, USA	Lava™ Ultimate (N895998)	80 wt. % (65 vol. %) nanoceramic particles (zirconia filler (4–11 nm), silica filler (20 nm), aggregated zirconia/silica cluster filler). 20 wt. % (35 vol. %) highly cross linked (methacrylate-based) polymer matrix. Silane		
Coltene, Cuyahoga Falls, OH, USA	Total Etch (H43207)	35% phosphoric acid		
Ivoclar vivadent, Schaan, Liechtenstein	Telio Onlay provisional restoration (Y51870)	The monomer matrix consists of monofunctional and difunctional methacrylates (36 wt. %). The fillers are highly dispersed silicon dioxide and copolymers (62.6 wt. %). Fluoride (1500 ppm), initiators, stabilizers and pigments (0.6 wt. %) are additional ingredients.		

HEMA: hydroxyethyl methacrylate; GPDM: glycero-phosphate dimethacrylate; PAMM: phthalic acid monoethyl methacrylate; TEGDMA: triethylene glycol dimethacrylate; UDMA: urethane dimethacrylate; bisGMA: bisphenol A-glycidyl methacrylate; YF: Ytterbium fluoride; CQ: camphorquinone; UV: ultraviolet; MDP: methacryloyloxydecyl dihydrogen phosphate; DDMA: dodecandiol-dimethacrylate; BHT: butylated hydroxytoluene; META: methacryloyloxyethyl-trimellitate-anhydride; DBP: dibenzoyl peroxide; BP: benzoylperoxide; EDTA: Ethylenediaminetetraacetic acid; wt. %: weight percentage; vol. %: volume percentage.

**Table 2:** Means ± standard deviations of micro-tensile bond strength (µTBS) (MPa) and number of beams (percentage) of each failure type.

	DC + H			LC		
	IDS1	DDS	IDS2	IDS1	DDS	IDS2
OptiBond FL/Maxcem (Kerr)	19.11 ± 9.32 **A,1,2** 11(24.4) *	21.70 ± 10.18 **A,3** 37(56.9) *	18.46 ± 10.67 **A,3 ** 37(50.6) *	26.75 ± 6.34 34(75.5) *	29.48 ± 9.44 27(41.5) *	27.59 ± 10.00 35(47.9) *
OptiBond Universal/Maxcem (Kerr U)	15.47 ± 7.48 **A,1** 38(67.8)	12.96 ± 9.79 **A,1** 26(74.2)	14.75 ± 6.19 **A,1,2** 44(67.6)	18.00 ± 6.94 17(30.3)	10.88 ± 4.97 9(25.7)	15.69 ± 10.43 16(24.6)
Prime& Bond active universal/Calibra Ceram (Dentsply)	20.16 ± 12.97 **A,1,2** 24(57.1) *	18.89 ± 10.78 **A,2,3** 25(64.1)	17.71 ± 7.57 **A,2,3** 37(64.9)	32.09 ± 10.01 15(35.7) *	24.91 ± 10.97 13(33.3)	21.86 ± 9.80 20(35)
Scotchbond Universal Adhesive/Relyx Ultimate (3M)	20.02 ± 10.56 **A,B,1,2** 25(46.2)	16.43 ± 11.03 **A,1,2** 51(73.9) *	24.47 ± 11.17 **B,4** 43(53.75) *	23.57 ± 9.10 27(50)	28.10 ± 12.75 18(26) *	33.19 ± 10.46 35(43.7) *
Future bond Universal single bond/Rebilda DC Cement (Voco)	14.80 ± 5.57 **A,B,1** 23(45)	13.52 ± 5.32 **A,1** 36(87.8)	17.43 ± 5.98 **B,3** 45(68.1)	17.69 ± 8.04 24(47)	12.47 ± 3.08 5(12.1)	16.47 ± 5.76 11(16.6)
Universal Primer Dual Cured Adhesive/Duo-Link (Bisco D)	8.51 ± 4.56 **A,B,3** 10(21.2)	8.57 ± 7.11 **A,1** 39(67.2)	11.30 ± 6.17 **B,1** 49(83)	8.74 ± 4.96 37(78.7)	9.30 ± 6.00 19(32.7)	12.88 ± 5.07 9(15.2)
All Bond Universal Agent/Duo-Link (Bisco U)	15.95 ± 9.01 **A,B,1** 41(75.9) *	17.39 ± 7.68 **A,2** 40(76.9)	13.64 ± 6,81 **B,1** 42(75) *	28.91 ± 19.26 12(22.2) *	18.72 ± 9.88 11(21.1)	18.17 ± 6.21 14(25) *
Ivoclar Adhese Universal/Variolink (Ivoclar)	24.37 ± 8.92 **B,2** 32(44.4)	19.17 ± 10.69 **A,2,3** 33(48.5) *	20.08 ± 8.71 **A,3** 43(55.1) *	28.77 ± 10.19 40(55.5)	26.51 ± 9.72 35(51.4) *	24.64 ± 8.55 34(43.5) *
One Coat 7 Universal/Solocem (Coltene)	24.32 ± 11.35 **B,2** 38(59.3)	15.69 ± 9.46 **A,1,2** 56(67.4)	19.58 ± 6.79 **B,3** 38(57.5)	23.76 ± 13.79 18(28.1)	17.19 ± 8.023 17(20.4)	23.35 ± 7.53 18(27.2)

Different letters in rows indicate significant differences between protocols in the same A-C. Different numbers in the same column indicate significant differences between A-C in the same adhesive strategy. * indicates significant differences between the types of failures in the same A-C and adhesive strategy.

## Data Availability

Data used in this publication is available upon request to the corresponding author.
